# Prevention and control of tuberculosis in workplaces: how knowledgeable are the workers in Bangladesh?

**DOI:** 10.1186/s12889-015-2622-4

**Published:** 2015-12-24

**Authors:** Qazi Shafayetul Islam, Md Akramul Islam, Shayla Islam, Syed Masud Ahmed

**Affiliations:** Bangladeshi-Canadian Community Services, Toronto, Canada; Health, Nutrition and Population Programme, BRAC, Dhaka, Bangladesh; Centre of Excellence for Universal Health Coverage James P Grant School of Public Health, BRAC University, 68 Shahid Tajuddin Ahmed Sharani, 5th Floor(Level-6), ICDDR,B Building Mohakhali, Dhaka, 1212 Bangladesh

**Keywords:** DOTS, Bangladesh, Workplace TB prevention and control, Chronic cough

## Abstract

**Background:**

The National Tuberculosis (TB) Control Programme (NTP) of Bangladesh succeeded in achieving the dual targets of 70 % case detection and 85 % treatment completion as set by the World Health Organization. However, TB prevention and control in work places remained largely an uncharted area for NTP. There is dearth of information regarding manufacturing workers’ current knowledge, attitudes and practices (KAP) on pulmonary TB which is essential for designing a TB prevention and control programme in the workplaces. This study aimed to fill-in this knowledge gap.

**Methods:**

This cross-sectional survey was done in multiple workplaces like garment factories, jute mills, *bidi*/tobacco factories, flour mills, and steel mills using a multi-stage sampling procedure. Data on workers’ KAP related to pulmonary TB were collected from 4800 workers in face-to-face interview.

**Results:**

The workers were quite knowledgeable about symptoms of pulmonary TB (72 %) and free- of-cost sputum test (86 %) and drug treatment (88 %), but possessed superficial knowledge regarding causation (4 %) and mode of transmission (48 %). Only 11 % knew about preventive measures e.g., taking BCG vaccine and/or refraining from spitting here and there. Knowledge about treatment duration (43 %) and consequences of incomplete treatment (11 %) was poor. Thirty-one percent were afraid of the disease, 21 % would feel embarrassed (and less dignified) if they would have TB, and 50 % were afraid of isolation if neighbours would come to know about it. Workers with formal education (AOR 1.92; 95 % CI 1.61, 2.29) and exposure to community health workers (CHW) (AOR 31.60; 95 % CI 18.75, 53.35) were more likely to have TB knowledge score ≥ mean. Workers with knowledge score ≥ mean (AOR = 1.91; 95 % CI:1.44, 2.53) and exposure to CHWs either alone (AOR = 42.4; 95 % CI: 9.94, 180.5) or in combination with print media (AOR = 37.35; 95 % CI: 9.1, 180.5) were more likely to go to DOTS centre for treatment . Only around 43 % had sputum examination despite having chronic cough of ≥ 3 weeks duration.

**Conclusion:**

The workers had inadequate knowledge regarding its causation, transmission and prevention which may interfere with appropriate treatment-seeking for chronic cough including sputum test. NTP needs to be cognizant of these factors while designing a workplace TB prevention and control programme for Bangladesh.

## Background

In 2003, the World Health Organization (WHO) and the International Labour Organization (ILO) highlighted workplace as an appropriate setting for initiating tuberculosis (TB) prevention and control activities [1]. This would also contribute in controlling TB in the communities since a person with infectious TB is estimated to infect about 20 people in his/her lifetime [1]. They emphasized the importance of workplace for implementing TB DOTS (Directly Observed Treatment, Short course) programme because “the workplace represents an opportunity for making TB control convenient and accessible to infected workers…and employers have the responsibility to implement measures that decrease the occupational risk of TB”.

The consequences of TB for workers are missed work, work disruptions, and reduced productivity [1, 2]. In 2006, Global Fund estimated that in the developing world, TB causes a loss in productivity amounting to US$12 billion per year as 75 % of TB deaths occur in persons in the economically productive age of 15 to 54 years [3]. In many instances, workers do not have access to the community-based TB DOTS programme during working hours and TB DOTS programme often provides no alternative for such workers [4]. In a study among 2281 workers from 30 garment factories in Dhaka city, the prevalence of TB was found to be higher (960 per 100,000) than in the general population [5]. Thus, manufacturing workplaces are of particular interest to national TB control programme (NTP), especially in high burden countries like Bangladesh which has a TB prevalence of 402 (all forms, PTB and non-PTB) and incidence of 224 per 100,000 per year in 2013 [6].

Bangladesh adopted community-based DOTS strategy for prevention and control of TB in 1993 and the NTP, through public-private partnership (PPP), succeeded in achieving the target of 70 % case detection and 85 % treatment success by 2003 as set by the WHO [7]. To expand TB prevention and control activities in areas not covered before, the NTP in its 5-year strategic plan (2006–2010) emphasized the involvement of business organizations in providing TB services in workplaces in line with WHO recommendations [2, 3].

In 2008 under a PPP (public-private partnership) model, NTP signed a MOU with the Bangladesh Garment Manufacturers and Exporters Association (BGMEA) to implement TB DOTS programme in 170 garment factories in Dhaka in association with the partner NGOs. The programme included advocacy and orientation activities among owners and workers of the garment factories, and TB management training for the medical officers and clinic managers of the BGMEA health centers. Besides, sputum test for chronic cough patients and supervised DOTS therapy for sputum positive cases were provided [8, 9]. Based upon experiences gained, the programme was later extended to BGMEA affiliated factories in Narayanganj and Gazipur areas near Dhaka, and ultimately it is planned to implement in all BGMEA affiliated factories in the country. Besides, DOTS centres were established by NTP in the hospitals of Dhaka and Chittagong Export Processing Zones (EPZ) and Youngone factory, through statutory body of the Bangladesh EPZ Authority (BEPZA) and implemented by partner NGOs (BRAC, Damien Foundation and *Progoti Samaj Kallyan Protisthan* PSKP) [9].

Workers engaged in garment factories (16 % of all manufacturing establishments) constitute only 55 % of the total workforce (5,015,936) engaged in manufacturing industries in Bangladesh in 2012 [10]. Beside experiences with some garment factories in and around Dhaka city, TB in other types of workplaces in other parts of the country remained largely an uncharted area for NTP in Bangladesh. However, for designing a multiple workplace TB prevention and control programme, information is needed from different manufacturing settings and this study aimed to fill-in this knowledge gap.

## Methods

### Settings

The study was done in six city corporation areas and respondents (workers) were recruited from five categories of manufacturing units such as garment factories (Dhaka and Chittagong), *bidi*/Tobacco factories (Rangpur and Barisal), steel mills (Chittagong), jute mills (Khulna) and flour mills (Sylhet). The factories/mills were selected respectively from areas where these are mainly concentrated. These factories/mills were medium in size, mostly employed around 1000 workers, and were situated in the neighbourhood of BRAC’s urban TB programme operational areas. BRAC is an indigenous NGO which works with the NTP to implement community-based DOTS programme in 297 out of 460 sub-districts in the country [11]. Given the problem of access during work hour, and constraints in time and resources, this approach facilitated logistic help from BRAC in selecting the factories/mills for the study and inviting the prospective respondents. Data were collected during February to March 2013.

### Sample and sampling technique

The sample size was determined on the basis of maximum proportion (0.5) with 5 % significance level and a precision of 5 %. The minimum calculated sample size for each category of factory/mills was 384 ~ 400 respondents with chronic cough of more than 3 weeks duration. To reduce design effects and keep provision for non-response, the sample size was increased to two times more i.e., 400 × 2 = 800 for each area. However, due to a sudden lay-out of the majority of the workers in the steel mills due to industrial unrest during the time of survey, we could not get our targeted number of samples and included all that were available and fulfilled our inclusion criteria (pl see below).

We used multi-stage sampling technique to draw samples from different manufacturing units mentioned above. Before sampling, lists of factories/mills were obtained from BRAC working in the above areas. From the list, eight manufacturing units were chosen purposively from each of the five areas (5 × 8 = 40) plus eight manufacturing units from each of Dhaka and Chittagong for garment factoriestotaling 56 units. Eight units were chosen because that was the minimum number of mills/factories adjacent to which BRAC TB programme was working at the time of the survey. For Garment factories and *bidi*/Tobacco factories, samples were taken from two areas so as to represent the two major areas of concentration of these two categories of factories/mills. Next, we aimed to include 100 workers from each factory/mill who had a history of cough of more than 3 weeks duration at the time of survey.

On the day of the survey, two interviewers were posted early in the morning at the point of entrance to the factory/mills who provided each worker with an identification number who had cough of ≥ 3 weeks duration and informed them about the survey including its purpose and methods. They were then requested to attend a face-to-face interview session (not to last more than 30 min) with the survey team at a time of their convenience e.g., during lunch time or scheduled breaks. The interviews were conducted in the workers’ native language i.e., Bangla.

### Data collection procedures

#### Questionnaire

A semi-structured questionnaire was used to collect data from the sampled workers. The questionnaire was developed in Bangla based on review of the literature on TB KAP studies and researchers’ own experiences. The questionnaire was pre-tested in areas outside the survey areas for consistency, culture-sensitive language, sequencing of questions, and insights into the field operation procedure. The draft questionnaire was modified and updated based on feedback from field-testing and finalized for the survey. Data were collected on socio-demographic profile of the respondents, their basic knowledge about TB including its diagnosis and treatment, and their health-seeking behaviour in relation to chronic cough.

#### Training

Twenty-five social science graduates with past experiences in survey data collection were recruited for the study through a competitive process. These interviewers received a 5-day intensive training consisting of didactic lectures, mock interviews, role play and field practice in workplaces. A training manual was developed to guide the interviewers in the field. Field trial was done outside the study areas under the supervision of the research team.

#### Data collection

Five teams were formed for data collection, each consisting of four interviewers and one supervisor. The teams were deployed in the workplaces 1 day before the survey began for rapport building with workers and administrative officers in the factories/mills. During this time, administrative officers were informed about the purpose and activities of the survey and the interviewers sought their cooperation for successful completion of the survey. The field activities were directly supervised by the researchers and due guidance was provided when needed. Whenever necessary, re-interview was done by the supervisors for securing reliable and valid data. To improve the latter, an independent quality control team spot-checked collected data randomly within 3 days of the main survey. In cases where inconsistencies were noted, interviewers were re-briefed on survey techniques and accompanied by the field supervisors until quality standards were met.

### Data processing and analysis

Data were cleaned, edited, and coded using the SPSSWIN (version 16.0) for analysis at Dhaka office of the BRAC Research and Evaluation Division under the supervision of the principal investigator. Mean was computed for continuous variables such as age and years of schooling while proportion was calculated for categorical variables. Appropriate statistical tests (*X*^2^ and *t*-test) were done where needed. Logistic regression was run to estimate Odds Ratios adjusted for age, sex, education, household economic status and exposure to TB information (AOR) with 95 % confidence intervals (CI).

For calculating knowledge score, one point was given for each correct answer to the eleven questions and overall knowledge score was calculated by summing these scores, the maximum score being 11 [12, 13]. The mean of the scores was taken as the cut-off point and respondents were categorized according to the level of score ‘at/above the mean’ and ‘below the mean’. Health-seeking behavior for TB was elicited with reference to management of cough of more than 3 weeks duration. Household’s poverty status was ascertained by self-reported economic status of the household in the past year and was categorized into ‘deficit’ (poor), ‘break-even’ and ‘surplus’ (least poor) households. Exposure was determined according to the source of TB-related information reported by the respondents.

### Ethical issue

The study passed through the institutional review process at BRAC Research and Evaluation Division (RED) for ethical approval. The study did not have any invasive procedure and information was collected through face-to-face interview. Since the respondents comprised largely of an illiterate/semiliterate community, and since the rural community in Bangladesh is largely skeptical of signing any written documents for historical reasons, written consent was not feasible. Instead, we opted for informed verbal consent from the respondents before conducting the interview. In this process, the written consent was read to the respondents with necessary explanation in the presence of a literate witness. When the interviewer was satisfied that the respondent understood it including its implications, and had agreed to participate, only then the respondent was included in the survey and interviewed.

## Results

Except for the steel mills, the total targeted sample from the four categories of factories/mills was 4800 of which interview could be completed for 4554 workers (response rate 95 %). In case of steel mills, all the available 246 respondents fulfilling the inclusion criteria were interviewed. In total. 4800 (4554 + 246) respondents were interviewed.

The mean age of workers from the five categories of factories/mills was 31 years, the youngest group coming from the garment factories (mean age 24 years) (Table 1). Males were in majority in flour (81 %), steel (69 %), and jute mills (64 %) while females were in majority in garment and *bidi*/tobacco factories. Mean years of schooling was four only and 1/3rd of the workers had no formal schooling. Forty-seven percent of the respondents self-rated their household economic status as poor (‘deficit’), the proportion reaching to 60 % in case of the respondents from the *bidi*/tobacco and flour mills. Around 9 % of the respondents stated that they had a TB patient in their households.

**Table 1:** Demographic and socioeconomic characteristics of the study workers

	Jute mill workers	Garment factory workers	Steel mill workers	Bidi factory workers	Flour mill workers	All
Mean age (±SD)^a^	37.5 (±13.0)	24.0 (±6.7)	33.5 (±11.3)	35.8 (±13.4)	29.8 (±12.2)	31.4 (±12.6)
Sex (%)						
Male	63.8	46.7	69.1	38.0	81.3	53.8
Female	36.2	53.3	30.9	62.0	18.7	46.2
Education (%)						
Had formal education	72.4	87.3	61.4	46.1	63.0	66.8
Had no formal education	27.6	12.7	38.6	53.9	37.0	33.2
Mean years of schooling (±SD)	4.8 (±3.6)	5.9 (±3.4)	3.9 (±3.7)	2.5 (±3.2)	3.6 (±3.4)	4.2 (±3.7)
Monthly mean income BDT (±SD)^b^	4800.0 (±2081.9)	6336.9 (±2602.9)	5155.2 (±2672.9)	2170.4 (±2058.6)	5071.4 (±2770.2)	4533.5 (±2924.2)
Self-rated perceived household economy status in last year (%)						
Deficit	53.4	24.0	46.7	60.3	60.5	47.3
Break-even	39.7	44.8	40.7	34.1	26.4	37.4
Surplus	6.9	31.2	12.6	5.5	13.0	15.3
Have TB patients at home	10.2	8.1	8.1	7.6	10.3	8.7
N	801	1485	246	1470	798	4800

^a^ *SD* standard deviation

^b^1US$ = 79.40 BDT (March 2013)

### Knowledge of TB

The workers were quite knowledgeable about the symptoms of TB (72 %), free-of-cost sputum test (86 %) and free-of-cost treatment (88 %), but had only superficial knowledge about the causative organism (4.4 %), mode of transmission (48 %) and prevention (11 %) (Table 2). Knowledge about duration of treatment and consequence of non-compliance such as multi-drug resistance was poor (43 and 11 % respectively). Only fifty-five percent knew about NTP including DOTS. The mean knowledge score was 4.7 (out of a maximum score of 11.0) and only 55 % of the respondents achieved score at or above the mean. *Bidi*/tobacco workers [adjusted OR (AOR) = 2.25 (95 % CI: 1.78, 2.84)] and steel mills workers [AOR =2.45 (95 % CI: 1.73, 3.48)] were more likely to have a knowledge score at or above the mean than the jute mill, garment, and flour mill workers (Table 3). Workers who had formal education were more likely to have knowledge score at or above the mean [AOR = 1.92 (95 % CI: 1.61, 2.29)] than those without formal education. Individuals who had exposure to community health workers (through interpersonal communication on TB) were more likely to know about TB (AOR = 31.6 (95 % CI: 18.8, 53.4) than those exposed to mass media only [AOR = 5.0 (95 % CI: 3.40, 7.39)], and even more so when exposed to both the community health workers and the mass media (AOR = 56.4 (95 % CI: 38.02, 83.70)].

**Table 2:** Knowledge on TB by type of workers (*N* = 4800)

Knowledge variables	Jute mills workers	Garment workers	Steel mills workers	*Bidi* workers	Flourmills workers	Total
Who can have TB (correct: TB is a disease of both male and female)	140 (17.5)	603 (40.6)	99 (40.2)	559 (38.0)	211 (26.4)	1612 (33.6)
Cause of TB (correct: germs)	16 (2.0)	121 (8.1)	5 (2.0)	52 (3.5)	18 (2.3)	252 (4.4)
Mode of transmission (correct: air borne through sneeze-cough)	309 (38.6)	851 (57.3)	139 (56.5)	706 (48.0)	303 (38.0)	2308 (48.1)
TB transmission prevention method (correct: BCG vaccine/do not spit here and there)	92 (11.5)	221 (14.9)	36 (14.6	141 (9.6)	34 (4.3)	524 (10.9)
Main symptom of PTB (correct: persistent cough of more than 3 weeks duration)	565 (70.5)	1018 (68.6)	191 (77.6)	1132 (77.0)	557 (69.8)	3463 (72.1)
Knowledge about sputum test (correct: sputum test is done at DOTS centre free of cost)	677 (84.5)	1245 (83.8)	215 (87.4)	1347 (91.6)	647 (81.1)	4131 (86.1)
Knowledge about treatment (correct: treatment is done at DOTS centre free of cost)	685 (85.5)	1298 (87.4)	220 (89.4)	1341 (91.2)	672 (84.2)	4216 (87.8)
Knowledge about standard treatment duration (correct: 6 months)	181 (22.6)	659 (44.4)	120 (48.8)	860 (58.5)	265 (33.2)	2085 (43.4)
Rules of taking medicine (correct: in front of health workers)	40 (5.0)	233 (15.7)	49 (19.9)	184 (12.5)	43 (5.4)	549 (11.1)
Knowledge about incomplete treatment (correct: multidrug resistance may occur)	35 (4.4)	119 (8.0)	38 (15.4)	269 (18.3)	63 (7.9)	524 (10.9)
Whether knew about National TB control Programme (NTP) (correct: yes)	447 (55.8)	572 (38.5)	113 (54.1)	1160 (78.9)	401 (50.3)	2713 (56.5)
Mean ‘Knowledge score’^a^ (±SD)	4.1 (±1.9)	4.7 (±2.4)	5.1 (±2.1)	5.3 (±2.0)	4.0 (±2.0)	4.7 (±2.2)
‘Knowledge score’ at or above the mean (≥ 4.7)				2621 (54.6)		

Numbers in parentheses indicate percentages

^a^Knowledge score obtained by assigning one score for each correct answer (range: 0–11)

**Table 3:** Knowledge score of workers on TB by socio-demographic and exposure factors

		Level of knowledge score (max 11) n (%)			
		at or above the mean (≥ 4.7)	below the mean (< 4.7)	Crude OR (95 % CI)	Adjusted OR^a^ (95 % CI)
Types	Jute mill workers	364 (45.4)	437 (54.6)	1.16 (0.95,1.42)	0.74 (0.57,0.95)
	Garment workers	749 (50.4)	736 (49.6)	1.42 (1.12,1.7)	1.81 (1.44,2.29)
	Steel mill workers	153 (62.2)	93 (37.8)	2.30 (1.71, 3.10)	2.45 (1.73,3.48)
	*Bidi*/tobacco workers	1021 (69.5)	449 (30.5)	3.17 (2.65, 3.79)	2.25 (1.78,2.84)
	Flourmill workers	465 (41.7)	333 (58.3)	1	1
Sex	Female	1242 (56.0)	976 (44)	1.11 (0.99, 1.25)	0.99 (0.85,1.16)
	Male	1378 (53.4)	1204 (46.6)	1	1
Education					
	Had formal education	1845 (57.5)	1362 (42.5)	1.43 (1.27,1.61)	1.92 (1.61,2.29)
	No formal education	775 (48.7)	818 (51.3)	1	1
Age (years)					
	> 54	182 (55.7)	145 (44.3)	1.14 (0.90,1.44)	1.45 (1.10,2.00)
	35–54	823 (59.3)	566 (40.7)	1.32 (1.16,1.50)	1.31 (1.10,1.57)
	15–34	1615 (52.4)	1469 (47.6)	1	1
Self-rated household economic status in last year					
	Surplus	392 (53.3)	343 (46.7)	0.98 (0.830,1.15)	1.40 (1.13,1.75)
	Break-even	1007 (56.1)	789 (43.9)	1.10 (0.97,1.24)	1.13 (0.96,1.33)
	Deficit	1221 (53.8)	1048 (46.2)	1	1
Exposure to TB information					
	Exposure to CHWs and mass media	1736 (83.6)	340 (16.4)	61.60 (41.94,90.47)	56.4 (38.02,83.70)
	Exposure to mass media	739 (34.2)	1423 (65.8)	6.27 (4.29,9.13)	5.01 (3.40,7.39)
	Exposure to CHWs	114 (72.6)	43 (27.4)	31.98 (19.26,53.11)	31.6 (18.75,53.35)
	No exposure to the above sources	31 (7.7)	374 (92.3)	1	1

*CHW* Community Health Worker

^a^OR adjusted for age, sex, education, household economic status and exposure to TB communication

### Attitudes towards TB

Fifteen percent of the respondents wanted to keep a possible diagnosis and subsequent treatment of TB confidential (Table 4). More than one-fifth (21 %) would be embarrassed if they have TB and would have less self-dignity, and another 23 % were afraid that the family would loss social benefits (e.g., old-age pension) if the relevant authority came to know about it. Around 28 % were not interested to talk to TB patients, and believed that community would socially avoid the family when they would come to know about it (29 %). Thirty-one percent of the workers were afraid of the disease, and 50 % were afraid of isolation if neighbours would come to know about it (Table 4).

**Table 4:** Attitudes towards TB by type of workers (*N* = 4800)

Attitudes related statement	Jute mills worker	Garment workers	Steel mills workers	*Bidi* /tobacco workers	Flour mills workers	Total
Would not disclose to others if would have TB	79 (9.9)	172 (11.6)	43 (17.5)	255 (17.3)	164 (20.6)	713 (14.9)
Would not inform others if would be undergoing TB treatment	71 (8.9)	188 (12.7)	46 (18.7)	246 (16.7)	165 (20.7)	716 (14.9)
Would be ashamed if would have TB	187 (23.3)	254 (17.1)	27 (11.0)	347 (23.6)	188 (23.6)	1003 (20.9)
Would think less of self if would have TB	140 (17.5)	379 (25.5)	53 (21.5)	220 (15.0)	221 (27.7)	1013 (21.1)
Thinks family would lose social benefit if relevant authority knows about TB	274 (34.2)	216 (14.5)	62 (25.2)	276 (18.8)	292 (36.6)	1120 (23.3)
Think social group would isolate family if knows about TB	305 (38.1)	157 (10.6)	59 (24.0)	502 (34.1)	308 (38.6)	1331 (27.7)
Would not like to talk to TB patients	271 (33.8)	292 (19.7)	75 (30.5)	503 (34.2)	230 (28.8)	1371 (28.6)
Would be afraid of TB patients	353 (44.1)	356 (24.0)	84 (34.1)	449 (30.5)	232 (29.1)	1474 (30.7)
Think others would isolate if know about TB	339 (42.3)	596 (40.1)	145 (58.9)	910 (61.9)	408 (51.1)	2398 (50.0)

Number in parentheses indicate percentages

### Health-seeking behaviour for chronic cough including sputum test

Only a minor proportion of the respondents (< 30 %) went to appropriate places (i.e., formal facilities for TB diagnosis and treatment in the public/NGO sector) for treatment of chronic cough of more than 3 weeks duration (Table 5). The proportion was greater among *bidi* /tobacco workers (23.5 %) compared to others. Workers who had ‘’knowledge score at or above the mean (AOR = 1.91; 95 % CI:1.44, 2.53) and exposure to community health workers either alone (AOR = 42.4; 95 % CI: 9.94, 180.5) or combined with exposure to media (AOR = 37.35; 95 % CI: 9.1, 180.5) were more likely to go to appropriate places compared to those who had knowledge score below mean and had no or limited exposure to mass media. Only around 43 % went for sputum examination despite suggestive symptom of TB (Table 6). Trends similar to treatment-seeking were observed for sputum testing as well.

**Table 5:** Health-seeking behavior of study workers who had chronic cough of ≥ 3 weeks duration by socio-demographic characteristics and exposure to TB information

		Health seeking-behaviour			
		Appropriate n (%)	Not appropriate n (%)	Crude OR (95 % CI)	Adjusted OR^a^ (95 % CI)
Types	Jute mill workers	47(5.9)	754 (94.1)	0.44 (0.31,0.63)	0.29(0.19,0.42)
	Garment workers	60 (4.0)	1425 (96.0)	0.29 (0.21,0.42)	0.35 (0.24,0.51)
	Steel mills workers	39 (15.9)	207 (84.1)	1.33 (0.89,1.98)	1.05 (0.66,1.65)
	Bidi workers	345 (23.5)	1125 (76.5)	2.16 (1.69,2.76)	1.16 (0.86,1.55)
	Flour mill workers	99(12.4)	699(87.6)	1.0	1.0
Sex	Female	309 (13.9)	1909 (86.1)	1.32 (1.11,1.58)	0.95(0.77,1.17)
	Male	281 (10.9)	2301 (89.1)	1.0	1.0
Education					
	Had formal education	350 (10.9)	2857 (89.1)	0.69 (0.58,0.82)	1.03 (0.83,1.29)
	No formal education	240 (15.1)	1353 (84.9)	1.0	1.0
Age (years)					
	> 54	44 (13.5)	283 (86.5)	1.29 (0.92,1.81)	0.71 (0.48,1.10)
	35–54	214(15.4)	1175 (84.6)	1.51 (1.26,1.81)	0.83 (0.69,1.03)
	15–34	332 (10.8)	2757 (89.2)	1.0	1.0
Perceived household economic status in last year					
	Surplus	58 (7.9)	677 (92.1)	0.64 (0.53, 0.77)	0.71 (0.57,0.88)
	Break-even	186 (10.4)	1610 (89.6)	0.48 (0.36,0.64)	0.94(0.67,1.32)
	Deficit	346 (15.2)	1923 (84.8)	1.0	1.0
Exposure to health workers or mass media for TB information					
	Exposure to health workers and mass media	520 (25.0)	1556 (75.0)	67.34 (16.72, 271.14)	37.35 (9.1180.51)
	Exposure to mass media	22 (1.0)	2140 (99.0)	2.07 (0.49,8.84)	1.7 (0.39,7.32)
	Exposure to health workers	46 (29.3)	111 (70.7)	83.5 (19.95, 349.36)	42.4 (9.94,180.5)
	No exposure to the above sources	2 (0.5)	403 (99.5)	1.0	1.0
Knowledge score					
	at or above the mean (≥4.7)	511 (19.5)	2109 (80.5)	6.44 (5.04,8.23)	1.91 (1.44,2.53)
	below the mean (<4.7)	79 (3.6)	2101 (96.4)	1.0	1.0

Appropriate = visit to formal facilities (DOTS centres) in the public/NGO sectors, not appropriate = visit to informal places/health care providers such as the village doctors and the salespersons/dispensers at drug retail outlets/drug shops

^a^OR adjusted for age, sex, education, household economic status and exposure to TB communication

**Table 6:** Factors influencing sputum test done for chronic cough (≥ 3 weeks) among study workers by socio-demographic and exposure characteristics

		Had sputum test			
		Yes	No	Crude OR (95 % CI)	Adjusted OR^a^ (95 % CI)
		n (%)	n (%)		
Types	Jute mill workers	466 (58.2)	335 (41.8)	1.2(0.98,1.46)	1.04 (0.84,1.30)
	Garment workers	373 (25.1)	1112 (74.9)	0.29(0.24,0.34)	0.24 (0.19, 0.30)
	Steel mills workers	60 (24.4)	186 (75.6)	0.27 (0.20,0.38)	0.19 (0.13,0.26)
	Bidi workers	741 (50.4)	729 (49.6)	0.87 (0.73,1.04)	0.54 (0.44,0.66)
	Flourmill workers	428 (53.6)	370 (46.4)	1	1
Sex	Female	952 (42.9)	1266 (57.1)	0.98 (0.88,1.10)	1.26 (1.10,1.44)
	Male	1116(43.2)	1466 (56.8)	1	1
Education					
	Had formal education	1390 (43.3)	1817(56.7)	1.03 (0.91, 1.16)	1.26 (1.08,1.47)
	No formal education	678 (42.6)	915 (57.4)	1	1
Age (years)	> 54	166 (50.8)	161 (49.2)	1.64 (1.30,2.06)	1.31 (1.00, 1.71)
	35–54	712 (51.3)	677 (48.7)	1.67 (1.47,1.90)	1.23 (1.05,1.43)
	15–34	1190(38.6)	1894(61.4)	1	1
Perceived household economic status in last year					
	Surplus	224 (30.5)	511 (69.5)	0.51 (0.42,0.60)	0.84 (0.68, 1.03)
	Break-even	795 (44.3)	1001 (55.7)	0.92 (0.81,1.04)	1.13 (0.98,1.30)
	Deficit	1049 (46.2)	1220 (53.8)	1	1
Exposure to TB information					
	Exposure to health workers and mass media	1206 (58.1)	870 (41.9)	6.87 (5.21, 9.04)	4.87 (3.58,6.63)
	Exposure to mass media	715 (33.1)	1447 (66.9)	2.44 (1.85, 3.22)	2.84 (2.11,3.81)
	Exposure to health workers	79 (50.3)	78 (49.7)	5.01 (3.34, 7.54)	3.28 (2.13, 5.07)
	No exposure to the above sources	68 (16.8)	337 (83.2)	1	1
Knowledge score	at or above the mean (≥ 4.7)	1390 (53.1)	1230 (46.9)	2.5 (2.22, 2.81)	1.88 (1.61,2.18)
	below the mean (< 4.7)	678 (31.1)	1502 (68.9)	1	1

^a^OR adjusted for age, sex, education, household economic status and exposure to TB communication

## Discussion

This study was done to fill-in current gaps regarding workers’ knowledge, attitude and practice about TB and their health-seeking behaviour for chronic cough. The information is expected to help NTP in designing a TB prevention and control programme for workplaces in Bangladesh. Findings reveal poor knowledge on different aspects of TB among a substantial proportion of the workers. A high level of misperception exists regarding TB and its prevention and management, including a stigmatized attitude towards TB and TB patients. The major sources of healthcare accessed for treatment of suspected TB cases were not appropriate, and majority of them did not go for sputum test despite having a history of chronic cough. These findings and its implications for a national workplace TB intervention in Bangladesh are discussed.

This study found that only 1/3rd of the respondents perceived TB affecting both males and females. Similar finding was also observed from other studies in Bangladesh, where majority of the respondents perceived it to be a disease of males only [8, 14]. This misperception may acts as an obstacle for female workers to seek timely care, as female gender in itself is found to adversely affect diagnosis and treatment in Bangladesh [15]. The study also found limited knowledge among workers about the causative organism of TB like other developing countries [12–14]. This is plausible as knowledge of TB is mainly based on cultural explanations (emic perspectives of illness) in developing countries which put the cause on ‘smoking’ or ‘cold air’ rather than ‘germs’ [13, 14] and as such, Bangladesh is no exception. However, lack of knowledge about causative organism may adversely affect attitude towards the disease and appropriate treatment-seeking [16]. The gaps in knowledge regarding the duration of standard TB treatment may affect compliance with treatment completion and could lead to emergence of drug resistance, which is already a problem in Bangladesh [17].

We found that a substantial proportion of the workers (43 %) in the study locations were unaware about the current national TB programme (including DOTS) in the country. One of the reasons may be lack of adequate interaction between health workers and manufacturing workers due to difficulty in talking to the workers during working hours when entry into the factory is restricted due to security reasons as well as reluctance of the management to give time-off during working hours for such interaction to take place [8]. However, the same study showed that this barrier can be overcome if the management can be motivated towards the benefit of DOTS treatment of TB cases in the factory settings. Again, majority of the workers being semi-literate (average schooling: 4 years), it was difficult for them to read and comprehend TB messages disseminated through posters and billboards. For this population, interpersonal communication with the health workers was the key source of information, which is consistent with what is also found in case of malaria communication [18].

Workers’ fear of social isolation due to contraction of the disease and associated stigma observed in this study is a common phenomenon worldwide [19–21]. Stigma combined with poor knowledge of TB delays its diagnosis and causes treatment non-adherence, and may act as an important barrier to service utilization. This has implications for programme implementation for household contacts as around 9 % of the study respondents reported to have had a TB patient in the household. Social support can help patients overcome these barriers along with multiple IEC (information, education and communication) intervention from the NTP.

It was observed that despite having symptoms suggestive of TB (e.g., chronic cough of more than 3 weeks), the workers did not intend to go for sputum test, may be due to the fact that the workers were not allowed to go outside [8] and the complexities of attending DOTS diagnostic services [22]. Low paid workers often go to informal providers, which is common with the poor population in this country [23]. Resulting delay in treatment initiation [24] and its consequences are damaging to personal well-being of the TB patients and the community as well. As the study showed, a combined approach using both interpersonal communication by the CHWs, and mass media would be more effective in increasing knowledge and appropriate treatment-seeking for TB.

This study used a multi-stage sampling from some selected trades and workplaces and as such, may not be representative of the workplaces in the whole country. However, it used samples drawn from the major manufacturing belts in the country including representation from different trades and thus, the findings give a fair idea about the current state of the workers’ knowledge, attitudes and practices related to TB. This study used only quantitative survey to elicit workers’ KAP regarding TB. However, for development of culture-sensitive communication strategies, more insights on their emic model of TB illness and stigmatized attitude towards TB including its socioeconomic implications are needed. These necessiate qualitative study approaches which couldn’t be done due to time and resource constraints. A third limitation is that data on work environment were not included which could have better contexualised the KAP data as this has direct impact on the health of the workers including TB.

Besides workers’ KAP on TB, implementing workplace TB programme also depends on factory management’s good understanding about TB and treatment protocols, and their subsequent commitment to become involved in the programme [2, 7]. To achieve this, effective and efficient collaboration between NTP, implementing partners (e.g., NGOs), and workplace management is essential. Other interventions requiring consideration would include time-off for workers for facilitating the screening of suspected cases and initiate DOTS; using cured TB patients for peer motivation for screening and treatment, and making the IEC campaign culture-sensitive and appropriate for the target audience, e.g., manufacturing workers in this case.

## Conclusion

Findings reveal that the workers studied had various misperceptions and stigma towards TB, and poor knowledge regarding its causation, transmission and prevention. This would interfere with TB case detection in workplaces including sputum test for chronic cough and appropriate treatment-seeking from DOTS centres. Thus, NTP needs to address these challenges and design appropriate IEC and motivation campaigns to overcome these barriers while expanding DOTS facilities in the workplaces.

## References

[1] Maher D, Boldrini F, Pathania V, Alli BO (2003). Guidelines on workplace TB control activities: The contribution of workplaces TB control activities to TB control in the community.

[2] World Bank (2003). Public-private partnerships for health: a review of best practices in the health sector.

[3] Harper C (2007). Tuberculosis, a neglected opportunity?. Nat Med.

[4] Bennoor KS, Hassan MR, Rahman MF, Mahmud AM, Hossain MA, Haque ME (2000). Tuberculosis among garments workers: magnitude of the problem in Bangladesh. Asian-Pac Newslett Occup Health Saf.

[5] Hassan MR, Bennoor KS, Rahman MF, Mahmud AM, Hossain MA, Habib GM (2005). Incidence of pulmonary tuberculosis in garments workers of Dhaka City, Bangladesh. Bangladesh Med Res Counc Bull.

[6] World Health Organization (2015). Global Tuberculosis Report 2015.

[7] National Tuberculosis Control Programme (NTP) (2013). Tuberculosis control in Bangladesh: Annual report 2013.

[8] Ullah ANZ, Huque R, Husain A, Akter S, Akter H, Newell JN (2012). Tuberculosis in the work place: developing partnerships with the garment industries in Bangladesh. Int J Tuberc Lung Dis.

[9] Islam MN. Engagement of workplace in TB care and control in Bangladesh. Available from: http://www.who.int/tb/careproviders/ppm/BangladeshPPMWorkplaceYoungone.pdf . Accessed 22 Dec 2015.

[10] Bangladesh Bureau of Statistics (BBS) (2013). Survey of manufacturing industries (SMI) 2012.

[11] BRAC Health Programme (2012). Annual Report 2012: Bangladesh tuberculosis control programme NGO component.

[12] Bati J, Leggese M, Medhin G (2013). Community’s knowledge, attitudes and practices about tuberculosis in Itang special district, gamella region, south western Ethiopia. BMC Public Health.

[13] Tolossa D, Medhin G, Leggese M (2014). Community’s knowledge, attitudes and practices towards tuberculosis in Shinile town, Somali regional state, eastern Ethiopia: a corss-sectional study. BMC Public Health.

[14] Islam QS, Ahmed SM, Islam MA, Chowdhury AS, Siddiquea BN, Husain MA (2014). Informal allopathic provider knowledge and practice regarding prevention and control of TB in rural Bangladesh. Int Health.

[15] Karim F (2009). Gender matters: Understanding of access barriers to community-based tuberculosis care in Bangladesh. PhD Thesis.

[16] Mesfin M, Tasew T, Tareke I, Mulugeta G, Richard M (2005). Community knowledge, attitudes, and practices on pulmonary tuberculosis and their choices of treatment supervisor in Tigary, Northern Ethiopia. Ethiopia J Health Dev.

[17] Rifat M, Milton AH, Hall J, Oldmeadow C, Islam MA, Husain A (2014). Development of multidrug resistant tuberculosis in Bangladesh: A case control study on risk factors. PLoS One.

[18] Ahmed SM, Hossain MS, Kabir M (2014). Conventional or interpersonal communication: which works best in disseminating malaria information in an endemic Rural Bangladeshi Community?. PLoS One.

[19] Somma D, Thomas BE, Karim F, Kemp J, Arias N, Auer C (2008). Gender and socio-cultural determinants of TB-related stigma in Bangladesh, India, Malawi and Columbia. Int J Tuberc Lung Dis.

[20] Courtwright A, Turner AN (2010). Tuberculosis and stigmatization: pathways and interventions. Public Health Rep.

[21] Thu A, Ohnmar H, Win H, Nyunt M-T, Lwin T (2012). Knowledge, attitudes and practice concerning tuberculosis in a growing industrialized area in Myanmar. Int J Tuberc Lung Dis.

[22] De Cuevas RMA (2012). The complexities of attending TB diagnostic services for adults in resource poor settings. PhD Thesis.

[23] Ahmed SM, Hossain MA, Chowdhury AMR, Bhuiya A (2011). The health workforce crisis in Bangladesh: shortage, inappropriate skill-mix and inequitable distribution. Hum Res Health.

[24] Sreeramareddy CT, Panduru KV, Menten J, den Ende JV. Time delays in diagnosis of pulmonary tuberculosis: a systematic review of literature. BMC Infectious Dis. 2009; doi:10.1186/1471-2334-9-91. 10.1186/1471-2334-9-91 PMC2702369 19519917

